# Modifications to rhesus macaque TCR constant regions improve TCR cell surface expression

**DOI:** 10.1371/journal.pone.0314751

**Published:** 2025-01-09

**Authors:** Lori V. Coren, Matthew T. Trivett, Jorden L. Welker, James A. Thomas, Robert J. Gorelick, Emek Kose, Taina T. Immonen, Kelli Czarra, Christine M. Fennessey, Charles M. Trubey, Jeffrey D. Lifson, Adrienne E. Swanstrom

**Affiliations:** AIDS and Cancer Virus Program, Frederick National Laboratory for Cancer Research, Frederick, Maryland, United States of America; Rutgers: Rutgers The State University of New Jersey, UNITED STATES OF AMERICA

## Abstract

T cell immunotherapy success is dependent on effective levels of antigen receptor expressed at the surface of engineered cells. Efforts to optimize surface expression in T cell receptor (TCR)-based therapeutic approaches include optimization of cellular engineering methods and coding sequences, and reducing the likelihood of exogenous TCR α and β chains mispairing with the endogenous TCR chains. Approaches to promote correct human TCR chain pairing include constant region mutations to create an additional disulfide bond between the two chains, full murinization of the constant region of the TCR α and β sequences, and a minimal set of murine mutations to the TCR α and β constant regions. Preclinical animal models are valuable tools to optimize engineering designs and methods, and to evaluate the potential for off-target tissue injury. To further develop rhesus macaque models for TCR based cellular immunotherapy, we tested methods for improving cell surface expression of rhesus macaque TCR in rhesus macaque primary cells by generating five alternative TCRαβ constant region constructs in the context of a SIV Gag-specific TCR: 1. human codon optimized rhesus macaque (RH); 2. RH TCR with an additional disulfide linkage; 3. rhesus macaque constant sequences with minimal murine amino acid substitutions; 4. murinized constant sequences; and 5. murinized constant sequences with a portion of the exposed FG loop in the β constant sequence replaced with rhesus macaque sequence to reduce potential immunogencity. Murinization or mutation of a minimal set of amino acids to the corresponding murine sequence of the constant region resulted in the greatest increase in rhesus macaque TCR surface expression relative to wild type. All novel TCR constructs retained the ability to induce production of cytokines in response to cognate peptide antigen specific stimulation. This work can inform the design of TCRs selected for use in rhesus macaque models of TCR-based cellular immunotherapy.

## Introduction

Adoptive Cell Therapy (ACT) using T cells has become a validated form of immunotherapy for a number of cancers since the first clinical experiments were conducted in the late 1980s with multiple products currently approved by the FDA [1–21]. T-cell based immunotherapies may consist of Chimeric Antigen Receptor (CAR) engineered cells, as well as Tumor Infiltrating Lymphocytes (TIL), or T Cell Receptor (TCR) selected or engineered cells [22]. The success of immunotherapy is reliant on myriad factors, one of which is the expression of the antigen receptor at the surface of the engineered or selected cell [23]. Given this, efforts have been made to optimize antigen receptor expression at the surface of engineered cells. In the case of TCR gene-modified cells, numerous approaches have been pursued, including choice of engineering method [24–28], optimization of coding sequence [29], and addressing the potential for TCR mispairing and/or competition for CD3 [30–34].

Transfer of an exogenous TCRαβ combination to a mature, TCR expressing T cell may result in mispairing of α and β chains between the exogenous and endogenous TCRs [35,36]. Besides possibly resulting in TCRs with mixed pairing of α and β chains that would be unable to recognize antigen at the cell surface due to mismatched variable regions, these incorrect TCRs could potentially elicit autoreactive immune responses [36,37], or compete with functional TCRs for CD3 complex formation [38–40]. To reduce the likelihood of mispairing and associated suboptimal outcomes, a variety of strategies have been tested to optimize pairing through manipulation of the TCR constant region sequence. In one approach, amino acids were exchanged between the α and β chains within the constant region interface [30]. This approach was successful in reducing the expression of mixed TCRs but did not result in T cells with higher functional avidity. In another approach, individual amino acids within both the α and β constant regions were mutated to cysteines to create an additional disulfide bond connecting the two chains [32,33]. This was also successful in reducing TCR mispairing and enhanced the functionality of engineered cells. A third approach involved addition of a leucine zipper at the C terminus of the α and β chain constant regions which significantly reduced mispairing compared to wildtype. A higher proportion of T cells expressing a leucine zipper modified TCR were bifunctional, as measured by expression of both intracellular IFNg and CD107a in response to antigen specific stimulation, compared to cells transduced with a wildtype TCR [41].

As a byproduct of other work, it was shown that exchanging the α and β constant regions for those of another species, namely mouse constant regions in place of human, increased the cell surface expression of the hybrid human/mouse TCRs relative to wild-type human TCRs [34,42]. This finding led to the idea of “murinization”, in which both constant regions of a human TCR are exchanged for the corresponding murine sequence to achieve improved surface expression and higher functional avidity [34]. While this strategy showed clear improvement of TCR surface expression, it raised an obvious concern about the potential of increased immunogenicity, due to the mixture of human and murine sequences. Additionally, prior work had shown the FG loop of the murine TCR β constant sequence is immunogenic in other hosts [43–45]. To address concerns about the risk of immunogenicity associated with incorporation of murine sequences, Sommermeyer and Uckert identified the minimal number of amino acids within the human constant region that could be selectively exchanged for those of the murine sequence to achieve a level of TCR expression comparable to the fully murinized version, while reducing the risk of immunogenicity [45].

Our laboratory has been evaluating the potential of T cell immune responses to suppress AIDS virus infection, using a nonhuman primate model of rhesus macaque infection with Simian Immunodeficiency Virus (SIV) and ACT of virus-specific TCR engineered T cells. We have shown that rhesus macaque primary T cells can be engineered by γ-retroviral vector transduction to express functional SIV-specific TCRs and that infusion of such engineered cells can impact viral replication dynamics [46]. Our approach utilizes a pMSGV1 retroviral vector with an MSCV LTR retroviral promoter that drives expression of the rhesus macaque TCR sequence, which consists of both TCR α and β variable segments (TRAV/TRBV) and the human codon optimized TCR α and β constant regions (TRAC/TRBC) [47]. Given that the previous work to optimize TCR expression through reduction of mispairing has all been conducted using human and murine TCRs, we tested previously identified manipulations of the TCR constant region in the rhesus macaque system. To do this we used a well characterized Mamu-A*01 restricted TCR specific for the SIV Gag peptide CTPYDINQM (CM9) [48] and constructed the following α and β constant region versions: 1, human codon optimized rhesus macaque [RH]; 2. a version of the RH TCR with an additional disulfide bridge [CYS] (as in [32,33]); 3. the rhesus macaque constant sequences with minimal murine amino acid substitutions (Rhesus Minimal Murine [RMM]) (as in [45]); 4. the murinized constant sequences [MC]; and 5. the murinized constant sequences with a portion of the exposed FG loop in the β constant sequence replaced with rhesus sequence, (Murine Minimal Rhesus [MMR]). These TCRs were compared head-to-head for cell surface expression and TCR triggered production of cytokines in response to CM9 peptide antigen specific stimulation, measured by intracellular cytokine staining. We also indirectly assessed the potential for mispairing for each novel TCR compared to RH.

Murinization or a minimal set of specific changes to the rhesus macaque TCR constant region sequences can increase transduction/expression efficiency of a virus specific TCR on engineered primary rhesus macaque T cells, as assessed by cell surface expression of the transduced TCR. These novel TCR constructs retain specificity and functionality as measured by production of cytokines in response to cognate antigen stimulation. This work has implications for the design of TCRs used in rhesus macaque models of TCR based cellular immunotherapy.

## Methods

### Generation of novel TCR constructs and γ-retroviral viral vectors

All retroviral vectors used for this study were generated using a previously described modified pMSGV1 backbone [49]. The MSGV Rhesus Acceptor Golden Gate (RH) which contains human codon optimized Rhesus macaque TCR constant sequences (Cβ1) [47] (Addgene plasmid #64271) and an unpublished MSGV Murine Acceptor Golden Gate (MC) vector that contains human codon optimized Mus musculus TCR constant sequences (Cβ2) containing a disulfide bridge to increase pairing and stability (Addgene plasmid #128176) were the parental vectors modified for these experiments [50].

The Rhesus TCR constant disulfide bridge (RH CYS) construct was generated by site directed mutagenesis of the human codon optimized rhesus (RH) vector to alter both TRAC aa 47 (T to C change), and TRBC aa 56 (S to C change).

The Rhesus Minimal Murine (RMM) vector was designed by altering the MSGV Rh Acceptor (RH) vector (Addgene plasmid #64271, [47]) TCR alpha constant (Rh TRAC) and TCR beta constant (Rh TRBC) sequences by switching out several rhesus amino acids for those used in the murine alpha and beta constants (Mu TRAC/Mu TRBC): TRAC aa 89–92 (T-E-S-V to S-D-V-P) and TRBC aa E17K, I21A, F132I, E135A, and Q138H. These changes were based on the sequence location of human (Hu) to murine substitutions as previously described by Sommermeyer and Uckert [45]. These sequences were human codon optimized and synthetically produced by GeneART (ThermoFisher, Waltham, MA). The RMM TCR constant cassette was ligated into modified pMSGV1 backbone between the PacI-NotI restriction endonuclease sites to produce MSGV RMM.

The Murine Minimal Rhesus (MMR) vector was an adaptation of the MSGV Mouse Acceptor (MC) vector (Addgene plasmid #128176) with replacement of a portion of the TRBC containing the FG loop with rhesus sequence in domain 3 as previously described by [45]. The MMR TCR constant cassette was ligated into the modified pMSGV1 backbone between the PacI-SnaBI restriction endonuclease sites to create MSGV MMR.

Once the all vectors were sequence confirmed, the Mamu A*01 restricted CM9.6 TRAV (GenBank Accession #HQ622178.1) and the CM9.6 TRBV (GenBank Accession # HQ622178.1) segments were inserted into each vector using a previously described Golden Gate method of TCR vector cloning [47]. No changes were made to the Mamu A*01 CM9.6 TCR α and β variable regions.

Retroviral vectors were generated by transfecting vector constructs into Phoenix-RD114 packaging cells as previously described, generating a stable vector producing line (Phoenix-RD114 cell line received from Dr. Hans-Peter Kiem) [51]. Culture medium supernatants from producer line cultures were used as the source of retroviral vector particles.

### Animal care and blood collection

Whole blood was collected from sedated rhesus macaques in EDTA Vacutainer tubes (BD Biosciences, Franklin Lakes, NJ) under a protocol (AVP-013) approved by the Institutional Animal Care and Use Committee of the National Cancer Institute, National Institutes of Health (NIH) in NIH-Bethesda facilities. NIH-Bethesda is accredited by AAALAC International and follows the Public Health Service Policy for the Care and Use of Laboratory Animals (Animal Welfare Assurance Number D16-00602). Animal care adhered to the standards outlined in the “Guide for the Care and Use of Laboratory Animals (National Research Council; 2011; National Academies Press; Washington, D.C.), in accordance with the Animal Welfare Act. Efforts were made to pair house macaques when possible. Primary enclosures consisted of stainless-steel primate caging provided by a commercial vendor. Animal body weights and housing in cages of appropriate dimensions were regularly monitored. Abnormalities noted during study procedures or during the regular care of the animals were brought to the attention of the veterinary staff. Overall dimensions of primary enclosures (floor area and height) met or exceeded the specifications of The Guide for the Care and Use of Laboratory Animals, and the Animal Welfare Regulations (AWRs). Further, all primary enclosures were sanitized every 14 days at a minimum, in compliance with AWRs. Primary enclosures were contained within animals’ rooms under light (12-hr light/12-hr dark light cycle), temperature, humidity, and airflow monitoring using building automated controls. Animals were fed commercial monkey chow, twice daily, with supplemental enrichment food items provided daily, including, but not limited to, fruit or other produce at least three times per week. Filtered, chlorinated water was available ad libitum. Animals were observed at least twice daily by trained personnel, including behavioral assessments. Environmental enrichment included provision of species appropriate manipulatives, and foraging opportunities, as well as auditory (music) and visual (video watching) enrichment multiple times per week. All clinical procedures, including administration of anesthesia and analgesics, were carried out under the direction of a laboratory animal veterinarian. Steps were taken to ensure the welfare of the animals and minimize discomfort of all animals used in the study. Animals were closely monitored daily for any signs of illness, and appropriate medical care was provided as needed. The ACUC approved humane endpoint criteria for the study included: 1) weight loss > 15% body weight in 2 weeks or 20% body weight in 2 months or 25% overall, 2) documented opportunistic infection, 3) persistent anorexia >3–5 days without explicable cause, 4) severe intractable diarrhea that was nonresponsive to standard treatment and resulted in dehydration and debilitation of the animal, 5) progressive neurologic signs (i.e., instability on the perch bar, head tilt, nystagmus, ataxia, stupor, or depression), 6) significant cardiac and/or pulmonary signs (i.e., dyspnea, open-mouthed breathing, severe, previously unrecognized, cardiac murmur especially if resulting in pulmonary edema), 7) persistent leukopenia (as a general guideline defined as <1000 cells/L) or thrombocytopenia (as a general guideline defined as <30,000 platelets/L), 8) progressive or persistent anemia (<20% hematocrit), 9) CD4 depletion or other signs of progressive immunosuppressive disease, 10) body condition score <1.5/5 with weight loss, 11) any other serious illness. In addition to these specific criteria, the decision to euthanize any animal rested with the professional judgment of the veterinary staff, including due to the culmination of signs not directly related to those enumerated above. No animals were euthanized during this study.

### Cell culture

Primary rhesus T cells were isolated from whole blood by Ficoll-Paque Plus (GE Healthcare, Chicago, IL) gradient centrifugation and cultured in “RPMI Complete” (RPMI 1640 medium (Gibco, ThermoFisher Scientific, Waltham, MA) supplemented with 10% (vol/vol) fetal bovine serum (R&D Systems, Minneapolis, MN), 1% 2 mM glutamine (Life Technologies, ThermoFisher Scientific, Waltham, MA) and 1% penicillin/streptomycin (100 mg/mL; Life Technologies, ThermoFisher Scientific, Waltham, MA)) with 50 IU/mL interleukin-2 (IL-2) (Peprotech, Cranbury, NJ). On the day of isolation, cells were stimulated with T cell activation/expansion beads coated with α-CD2/3/28 antibodies prepared according to manufacturer’s instructions (5 μL/106 cells, Miltenyi Biotec, Gaithersburg, MD) and 50 IU/mL of IL-2. Cultures were maintained at 37°C and 5% CO_2_. Transductions were performed 48 hr after the initial bead stimulation.

### Transduction

Transduction of primary rhesus macaque peripheral blood mononuclear cells (PBMC) with CM9 RH, RH CYS, RMM, MC, and MMR TCR retroviral viral vector supernatants was carried out as previously described [52]. For experiments in which cells were transduced with equivalent amounts of quantitated vector, 5mL of previously frozen supernatant containing 2.3x10^9^ vector particles were loaded onto retronectin (TaKaRa Bio USA, San Jose, CA)-coated six-well non-tissue-coated plates (treated with 20 μg/mL) and centrifuged at 1300xg for 2 hrs 10 min at room temperature (RT) in a Beckman Coulter Allegra X-15R centrifuge. The supernatant was removed and 4x10^6^ rhesus primary macaque cells were added to each well. Rhesus primary macaque cells had been stimulated 2 days prior with T cell activation/expansion beads coated with α-CD2/3/28 antibodies prepared according to the manufacturer’s instructions (5 μL/10^6^ cells, Miltenyi Biotec, Gaithersburg, MD). The cells were then centrifuged at 400xg for 15 min at RT followed by incubation at 37°C with 5% CO_2_ for 48 hours before flow cytometric analysis for TCR expression. To assess mTCRβ antibody binding and intracellular cytokine assays 5 mL of each untitered fresh supernatant were loaded onto retronectin coated six-well non-tissue coated plates and transduction was carried out as described above.

### Flow cytometry: Cell surface staining and sorting

48 hrs after transduction cells were surface stained for 25 min at room temperature in the dark (RTD) for CD3 (20 μg/mL, SP34-2, BD Biosciences, Franklin Lakes, NJ), CD4 (20 μg/mL, OKT4, BD Biosciences, Franklin Lakes, NJ), CD8α (20 μg/mL, SK1, BD Biosciences, Franklin Lakes, NJ), mouse TCRβ (5μg/mL, H57-597, ThermoFisher Scientific, Waltham, MA), and CM9 tetramer (2μL, MBL International, Woburn, MA). Cells were washed (400xg, 8 min) in 4 mL D-PBS (Gibco, ThermoFisher Scientific, Waltham, MA) and supernatant was discarded. Cells were resuspended in 150 μL of D-PBS and data were immediately acquired on a BD Biosciences LSR-II or LSRFortessa X-20 (BD Biosciences, Franklin Lakes, NJ).

Six days post transduction primary rhesus macaque cells from one animal were surface stained for CM9 TCR using CM9 tetramer (2μL, MBL International, Woburn, MA). Tetramer positive cells were sorted into 12x75mm tubes on a Bigfoot cell sorter (ThermoFisher Scientific, Waltham, MA) using the “Purity” sort setting into three populations: CM9 negative, CM9 dim, and CM9 bright. Sorted populations were washed and pelleted for cell associated retroviral vector quantification.

### Intracellular cytokine assay

TCR transduced primary rhesus macaque PBMC were surface stained (see “*Staining for Flow Cytometry*”) and assessed for intracellular cytokine production in response to antigen specific stimulation 7 days after transduction. K562 antigen presenting cells [53] transduced to express Mamu A*01 MHC were pulsed with 1 μg/mL Gag CM9 peptide (Biosynth International, Gardner, MA) for 30 min at 37°C, 5% CO_2_. After washing unbound peptide away, antigen presenting cells were mixed with transduced cells at a 1:1 ratio, incubated for 6 hours at 37°C, 5% CO2 in the presence of BD Golgi Stop containing monensin (0.6 μL/test, BD Biosciences, Franklin Lakes, NJ) and CD107a antibody (75 μg/mL, H4A3, Biolegend, San Diego, CA) in a Digitherm incubator (Tritech Research, Los Angeles, CA), and then chilled to 4°C until staining and analysis. Cells were surface stained for 25 min RTD for CD3 (20 μg/mL, SP34-2, BD Biosciences, Franklin Lakes, NJ), CD4 (20 μg/mL, OKT4, BD Biosciences, Franklin Lakes, NJ), and CD8α (20 μg/mL, SK1, BD Biosciences, Franklin Lakes, NJ). Cells were washed (400xg, 8 min) in 4 mL D-PBS (Gibco, ThermoFisher Scientific, Waltham, MA) and supernatant was discarded. Cells were fixed and permeabilized using 0.5 mL BD Fix/Perm solution (BD Biosciences, Franklin Lakes, NJ) for 25 min RTD. Cells were suspended in an additional 3.5 mL of 1X BD Perm Wash (BD Biosciences, Franklin Lakes, NJ), for 5 min at RT and washed (550xg, 8 min). Antibodies to intracellular cytokines IFN-γ (25 μg/mL, B27, Biolegend, San Diego, CA), TNFα (35 μg/mL, Mab11, Biolegend, San Diego, CA), and MIP-1β (35 μg/mL, D21-1351, BD Biosciences, Franklin Lakes, NJ) were diluted in BD Perm Wash and incubated with cells for 25 minutes at 4°C in the dark. Cells were then washed twice in 4 mL of BD Perm Wash (400xg, 8 minutes) and resuspended in 150 μL of BD Perm Wash before immediate data acquisition on a BD LSR-II. Analysis was carried out in FCS Express (DeNovo Software, Pasadena, CA).

### Quantitation of retroviral vectors

To quantify retroviral vector in RD114 producer line supernatants, RNA from 10 μL of supernatant was isolated as previously described [54], with the exception that vector particles were not pelleted prior to lysis, but were lysed directly. Isolated RNA was subjected to cDNA synthesis as described in [54]. DNA contamination was monitored by a no-reverse transcriptase (RT) control. For quantitation of cell associated retroviral vector in transduced cells, DNA was isolated using TriReagent (Molecular Research Center, Cincinnati, OH) essentially as described in [55].

The cDNA (from supernatant samples) or DNA (from cell samples) was then directly quantified by droplet digital PCR (ddPCR) on a Bio-Rad QX200 ddPCR system using the BioRad (Hercules, CA) ddPCR Supermix for Probes (No dUTP) according to manufacturer’s instructions. MLV Gag primers and probe were obtained from Integrated DNA Technologies (Coralville, IA) with the sequences: Forward 5’GAGACGTTGGGTTACCTTCTG3’; Reverse 5’CCTTGATCTTAACCTGGGTGAT3’; Probe 5’/56-FAM/CGA GAC GGC /ZEN/ACC TTT AAC CGA GAC /31ABkFQ/3’. Thermocycling of the ddPCR droplets was 95°C, 10 min.; 40 cycles of 95°C, 30 sec. and 60°C, 1 min.; 98°C, 10 min.; 12°C hold. Data analysis was performed using QX Manager Standard Edition, v1.1 software. Results for cell associated vector copy number were normalized to diploid genome equivalents by quantitation of DNA copies for a single copy sequence from, as described [56].

The MLV Gag ddPCR assay was validated using EcoRI-linearized, gel purified plasmid MSGV RH without variable TCR inserts that was quantitated by Qbit (Thermo Fisher, Waltham, MA). Additionally, an RNA transcript was generated by digesting the MSGV MC plasmid with SpeI and EcoRI, the fragment (2252 bp) was gel purified and inserted in pZero (ThermoFisher, Waltham, MA). The resulting plasmid was linearized with Tth111I (New England Biolabs, Ipswich, MA) and transcript standards (641bp) were derived with Ribmomax SP6 kit (Promega, Madison, WI) and quantitated by A260. This transcript was subjected to cDNA synthesis as described above and used for ddPCR assay validation.

### Statistics

We used mixed effects methods to model the linear relationship between the amount of cell associated vector and transduction efficiency as measured by CM9 tetramer staining and for vector copy per cell and MFI, using lme package in R (version 4.1.1 2021-08-10) [57]. Mixed effects models combine population-level parameters, called fixed effects with added randomness to one or more parameters and thus, are better able to capture the variability in each individual. We observed significantly different slopes between donor animals when log-transformed MFI and CM9 transduction efficiency were were plotted against vector copy per cell, where the relationship was clearly linear. To account for inter-individual variability, we added random effects to the slope when expressing log-transformed MFI amd CM9 as linear functions of the vector copy per cell, for each animal. Random effects are normally distributed, with mean 0 and standard deviation ω_m_. The random effects are only placed on the slope parameter, which is lognormally distributed. We used the lmerTest package in R to calculate the confidence intervals for the fixed variables and the standard deviation for the random effects [58]. Cohen’s *f*^2^ was used to report the effect size for our linear mixed effects models, and is a function of marginal *R*^2^; $f^{2}=\frac{R^{2}}{1-R^{2}}$ [59]. To calculate *R*^2^ for the fixed effects of the model we used the performance package [60].

## Results

### Construction of novel RH CYS, RMM, MC, and RMM TCRs

**Fig 1:**
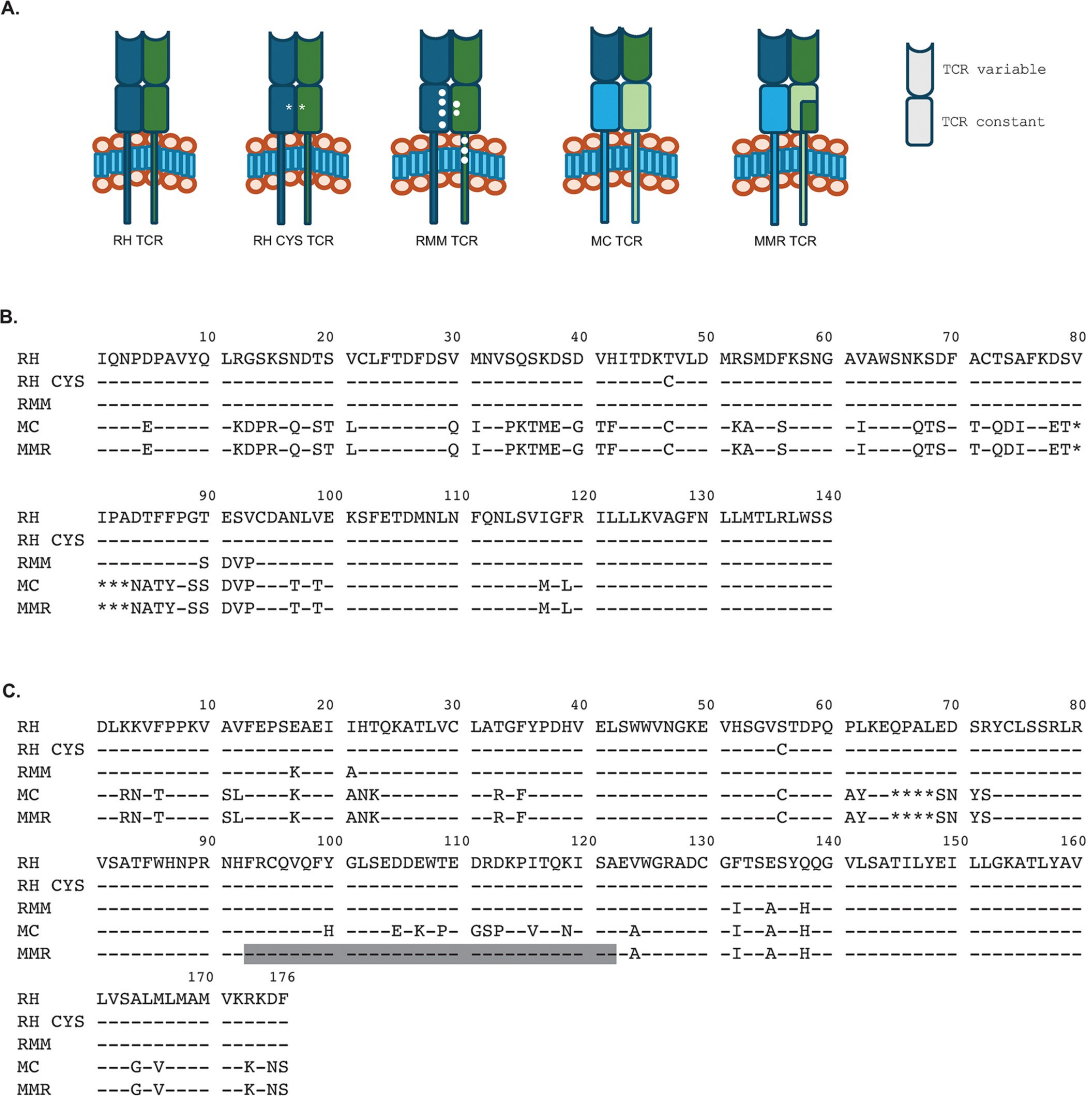
TCR construct design. **A**. Schematic of TCR constructs. Dark blue and dark green indicate rhesus macaque sequences. Light blue and light green indicate murine sequences. Asterisks indicate general location of introduced cysteines. Circles on RMM CR indicate general location of minimal murine mutations. TCR constant region alpha (**B**) and beta (**C**) amino acid sequences for all constructs. Gray box indicates region of FG loop. Numbering is based on rhesus macaque sequence.

To test the impact of modifications to the rhesus macaque TCR constant region on surface expression, functionality, and mispairing we developed four TCR constructs with manipulated constant regions for comparison with the unmanipulated sequence version of the SIV Gag CM9 specific rhesus macaque TCR with human codon optimized rhesus macaque α and β constant region sequences. To replicate earlier work showing that an additional disulfide bond between the α and β constant region chains may reduce TCR mispairing, we changed TRAC aa 48 (T) and TRBC aa 57 (S) to cysteines based on corresponding amino acids in the human reference sequence [33,61] (“RH CYS” Fig 1). To create “rhesus minimal murine” (RMM) constant regions, we mutated TRAC aa 89–92 (T-E-S-V to S-D-V-P) and TRBC aa E17K, I21A, F132I, E135A, and Q138H based on the findings of Sommermeyer and Uckert [45] (Fig 1). The murinized (MC) TCR was constructed by replacing the entire rhesus macaque TRAC and TRBC sequences with those of murine coding sequence with the addition of cysteines at TRAC aa48 and TRBC aa 57 (Fig 1). The “mouse minimal rhesus” (MMR) constant regions were based on the murine TRAC and TRBC sequences used in the MC construct, with TRBC aa93-122 replaced with the corresponding rhesus macaque sequence to remove a documented immunogenic sequence (Fig 1).

### Surface expression of novel TCRs

**Fig 2:**
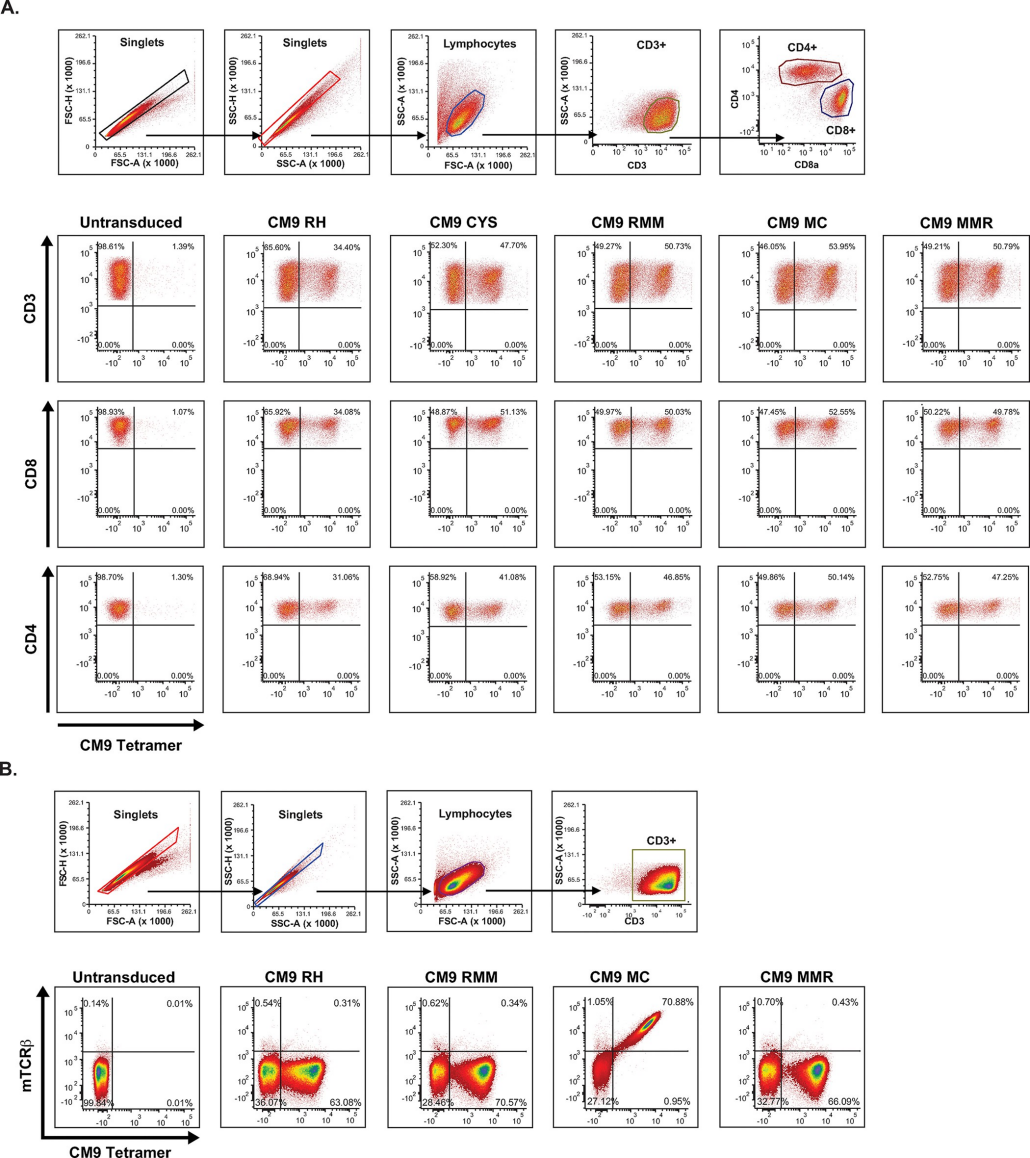
Surface expression of TCR construct. **A**. Gating strategy and expression of CM9 RH, RH CYS, RMM, MC, and MMR transduced TCRs on the surface of primary rhesus macaque CD3+, CD8+, and CD4+ T cells measured by CM9 tetramer staining. **B**. Gating strategy and staining of primary rhesus macaque CD3+ PBMC transduced with CM9 RH, RMM, MC, and MMR with a species-specific antibody to the murine TCRβ constant region (mTCRβ). One representative experiment shown.

The TCR constructs were cloned into our γ-retroviral vector and transduced into the rhesus macaque primary blood mononuclear cells (PBMC) using untitered fresh vector supernatant to assess surface expression (Fig 2A). The CM9 TCR expressed at the cell surface was specifically identified by pMHC tetramer staining. All TCR constructs were expressed at the cell surface, as evidenced by CM9 tetramer staining (Fig 2A), confirming that all transduced TCR constructs were able to integrate, be transcribed and translated, and be expressed in the correct heterodimeric form with CD3 at the cell surface. TCR expression was similar between the total CD3+ population and the CD8+ and CD4+ T cell subsets. Additional staining of the RH, RMM, MC, and MMR TCR transduced CD3+ PBMCs was performed with an antibody specific for the murine TCRβ constant region (Fig 2B). This confirmed that the exchange of rhesus macaque sequence for the murine in TRBC aa93-122 ablated the antibody binding site, as expected.

### In vitro functional evaluation of T cells transduced with RH, RH CYS, RMM, MC, and MMR TCRs

**Fig 3:**
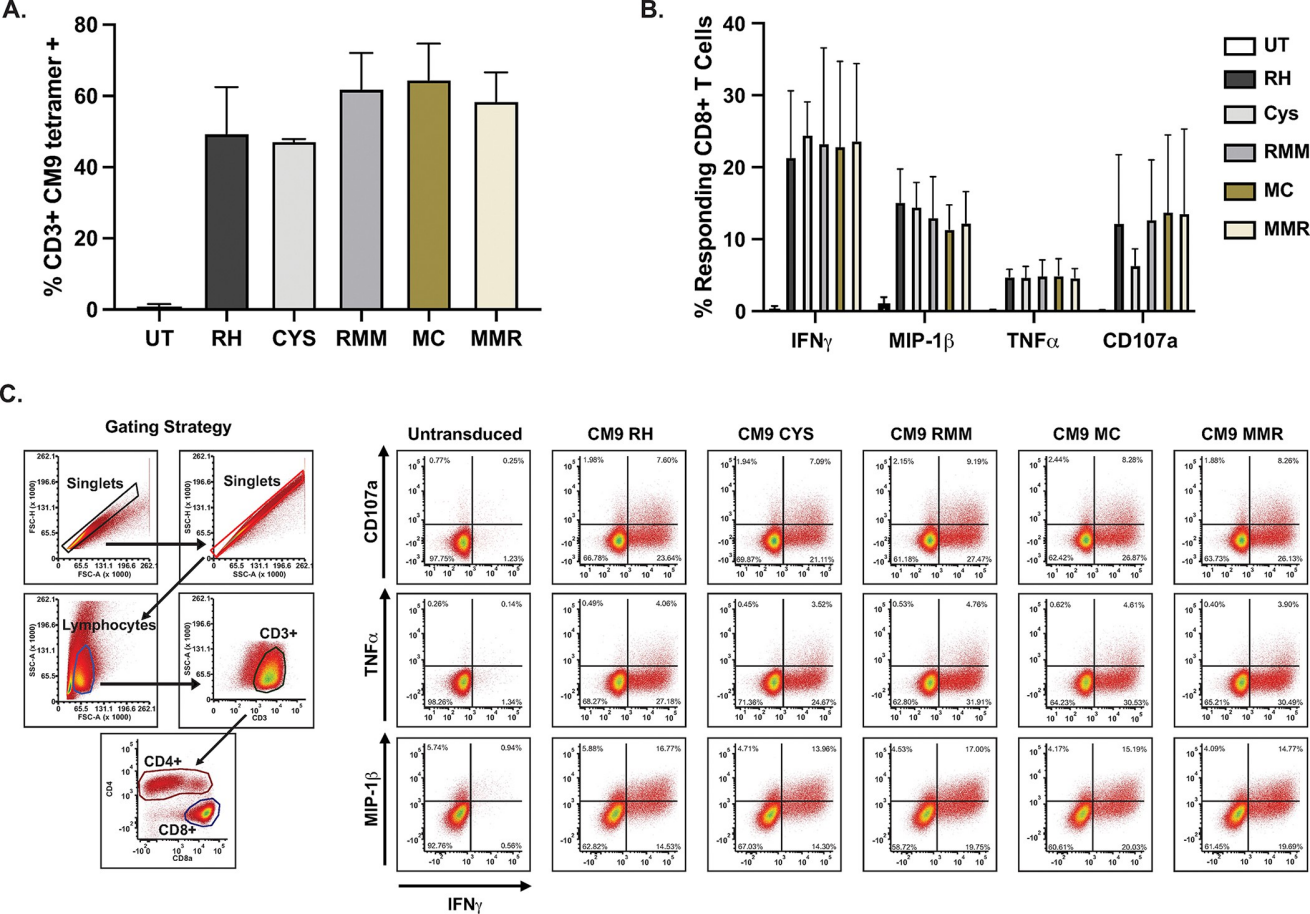
Cytokine responses of transduced cells. **A**. Expression of CM9 RH, RH CYS, RMM, MC, and MMR transduced TCRs on the surface of primary rhesus macaque cells used in the ICS assay as measured by CM9 tetramer staining. Data shown are the average and standard deviation of three experiments using transduced primary cells from three different rhesus macaques. **B**. Percentage of CD8+ T cells that produced IFNγ, MIP-1β, TNFα, or CD107a in response to CM9 peptide stimulation. Data shown are the average and standard deviation from three experiments using transduced primary cells from three different rhesus macaques and are background (no peptide loaded) subtracted. **C**. CM9 RH, RMM, MC, and MMR TCR transduced CD3+ CD8+ T cells produced IFNγ, MIP-1β, TNFα, or CD107a in response to CM9 peptide stimulation. One representative experiment shown.

To confirm that the TCRs expressed at the cell surface were functional, we performed an intracellular cytokine staining (ICS) flow cytometry assay (Fig 3). Rhesus macaque PBMCs were transduced with untitered fresh vector supernatant for each TCR construct, which resulted in approximately equivalent percentages of cells expressing the CM9 TCR based on CM9 tetramer staining (Fig 3A). Untransduced and transduced cells were stimulated with artificial antigen presenting K562 cells expressing rhesus macaque Mamu-A*01 (MHC-Ia) loaded with the cognate SIV Gag CM9 peptide. CD8+ T cells transduced with each of the TCR constructs produced the cytokines IFNγ, MIP-1β, TNFα, and the degranulation marker CD107a at similar frequencies (Fig 3B and 3C). These results show that our alteration of the TCR constant regions did not have a demonstrable impact on this TCR-dependent functional response, as measured by cytokine production in response to antigen specific stimulation.

### Indirect assessment of TCR mispairing

**Fig 4:**
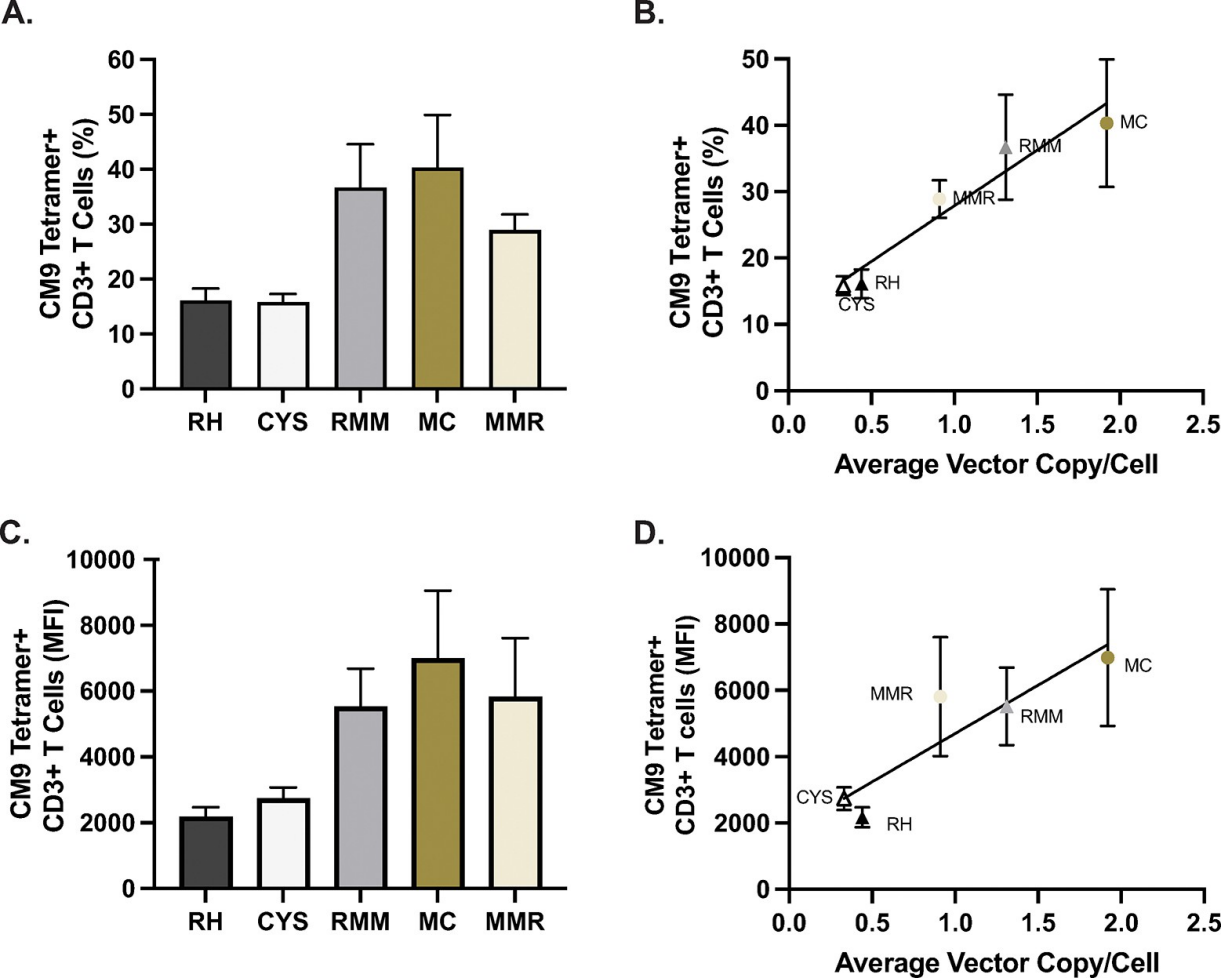
Relationship between TCR surface expression and cell associated γ-retroviral vector. **A**. Percentage of CD3+ transduced cells for each CM9 TCR construct that were positive for CM9 tetramer staining. **B**. Relationship between the percentage of CD3+ CM9 tetramer positive cells and the average vector copy per cell. **C**. Mean fluorescence intensity (MFI) of CM9 tetramer staining on transduced CD3+ cells for each CM9 TCR construct. **D**. Relationship between MFI and the average vector copy per cell. Data shown are the average and standard deviation of three experiments using transduced primary cells from three different rhesus macaques.

To indirectly assess whether TCR mispairing occurred more frequently in rhesus macaque PBMCs transduced with rhesus TCRs than in cells transduced with murinized or chimeric TCRs we transduced cells with equivalent numbers of vector virions as measured by ddPCR. Replicate experiments showed that the murinized TCR had the highest level of effective transduction efficiency, as reflected by the percentage of cells expressing the transduced TCR (Fig 4A). The RMM TCR construct was expressed at the cell surface in a similar, but slightly lower percentage of the population, than the MC construct. The mouse minimal rhesus (MMR) construct exhibited lower values than the MC and RMM constructs, but greater than that of RH and CYS. Results for the RH and CYS constructs were similar to each other, but approximately half that of the MC construct (Fig 4A). The results of the previous experiments (Figs 2 and 3) suggest that differences between TCR construct transduction efficiency can be overcome by the amount of vector used to transduce cells, which is indicative of mispairing as more copies of the exogenous TCR would increase the likelihood of successful pairing.

However, cell surface expression of a transduced TCR could be affected by differences in successful vector integration and at many of the post-integration processes required for correct assembly at the cell surface, including those that our modifications of the TCR constructs were intended to address. To address this question, we assessed the average vector DNA copy per cell in the bulk cell population by quantitative reverse-transcriptase PCR (qRT-PCR). Using a linear mixed effects model with random effects on the slope for each animal, we found a significant linear relationship (fixed effect slope = 0.27, CI = (0.18–0.36), p = 5.4E-05) between the amount of cell associated vector and transduction efficiency as measured by CM9 tetramer staining (Fig 4B, Table 1). To estimate a standardized effect size for the proportion of variance explained by the model relative to the unexplained variance, we computed Cohen’s f^2 based on the marginalized residual sum of squares for the fixed effect [59]. The overall effect size of the regression model was large (Cohen’s f^2 = 4.19).

**Table 1:** 

			**Standard Error**	**p-value**	**95% Confidence Interval**	**Rhesus Macaque 1**	**Rhesus Macaque 2**	**Rhesus Macaque 3**
**% CM9 Tet+**	**Fixed Effects**					**Individual Fits**		
	Slope	0.27	0.035	5.40E-05	(0.18–0.36)	0.27	0.3	0.23
	Intercept	1.14	0.037					
	**Standard Deviation of the Random Effects**							
	Slope	0.04						
**MFI**	**Fixed Effects**					**Individual Fits**		
	Slope	0.27	0.06	0.001	(0.14–0.42)	0.24	0.34	0.25
	Intercept	3.35	0.06					
	**Standard Deviation of the Random Effects**							
	Slope	0.06						

We also analyzed the mean fluorescence intensity (MFI) of CM9 tetramer staining on transduced cells, as a proxy for the relative number of TCR molecules at the cell surface. The results exhibited a similar pattern as for the frequency of CM9 tetramer positive cells, wherein the MC construct had the highest average MFI, the RMM and MMR constructs had slightly lower average MFIs that were similar, and the RH and CYS constructs had the lowest average MFIs (Fig 4C). Again, we found a significant linear relationship between log-transformed MFI and vector copy per cell (fixed effect slope = 0.28, CI = (0.14–0.42), p = 0.001), using a linear mixed effects model with random effects on the slope for each animal (Fig 4D, Table 1). Cohen’s *f*^2^ implies a large effect (*f*^2^ = 1.54).

**Fig 5:**
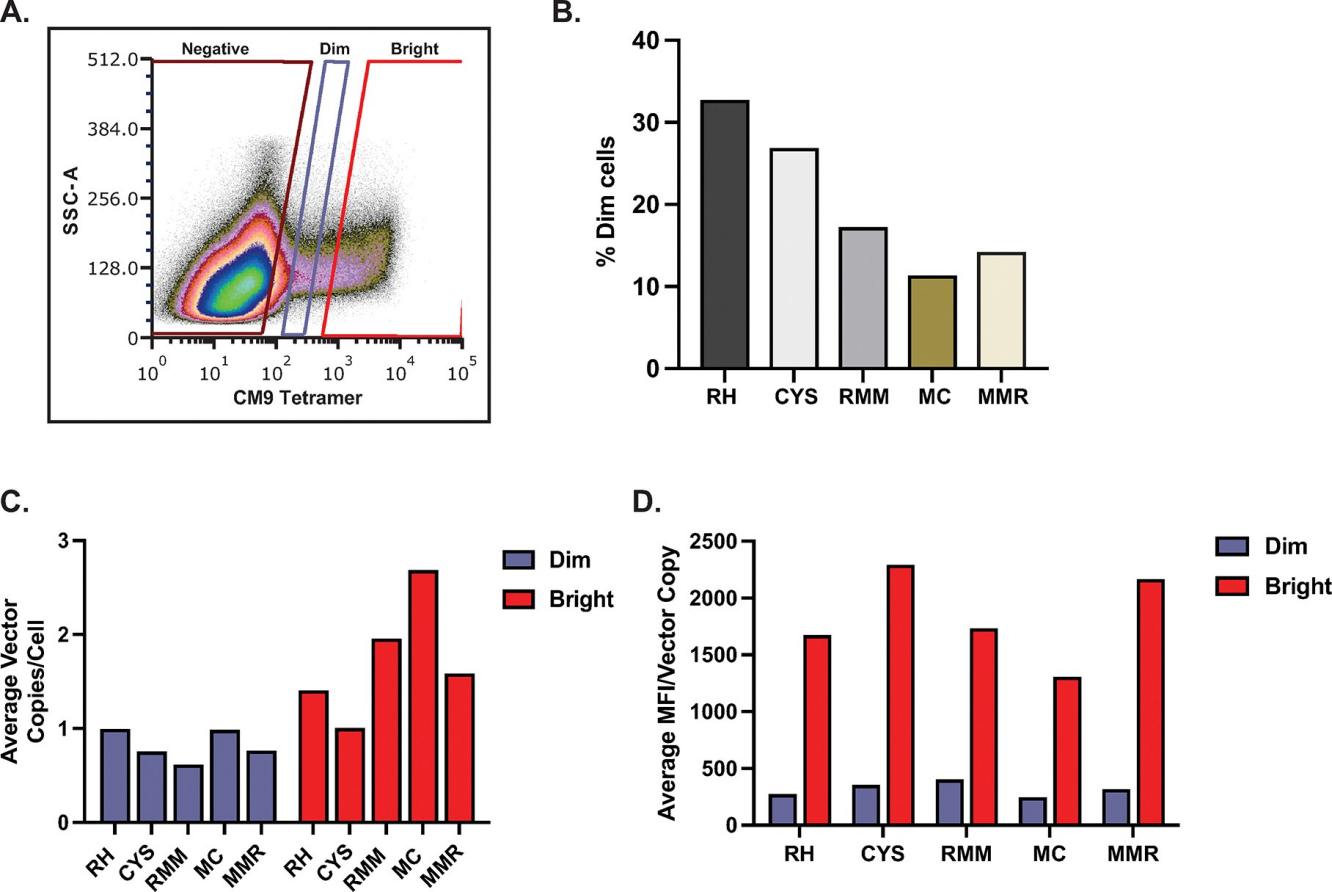
Analysis of γ-retroviral vector copies in cell populations sorted based on TCR expression. **A**. Example of gating used to isolate CM9 TCR negative, dim, and bright populations. SSC-A, side scatter area. **B**. Percentage of CM9 tetramer positive cells that fell within the dim gate. **C**. Average vector copies per cell in dim and bright sorted populations for each CM9 TCR construct. **D**. Average MFI normalized to cell associated vector copy in dim and bright sorted populations. Data shown are from one experiment.

The previous experiments were performed on bulk cell populations that included both transduced cells and untransduced cells since our transductions are not 100% efficient. This impacts the calculation of cell associated vector, as the vector quantities are averaged over the entire population, not just transduced cells. To indirectly, but more specifically, assess potential mispairing of transduced TCR with endogenous TCR, we used CM9 tetramer staining to sort primary rhesus macaque cells from one transduction experiment into negative, dim, and bright MFI populations based on the MFI of CM9 tetramer staining (Fig 5A). We hypothesized that the RH TCR transduced population would have a greater proportion of cells in the dim MFI population, as mispairing would result in fewer correctly paired transduced CM9 TCRs being expressed at the cell surface. When calculated as a percentage of all cells in the dim and bright gates, the RH construct did show the highest proportion of cells in the dim gate relative to the other constructs (Fig 5B). The MC construct had the lowest proportion of cells in the dim gate, with approximately 3 times fewer cells in this gate than the RH construct (Fig 5B). Similarly, we hypothesized that the RH TCR bright population would have a higher average number of vector copies per cell than the other constructs, because more cell associated vector copies may be required to overcome TCR mispairing to achieve a comparable level of cell surface expression of the transduced TCR. Quantification of cell associated vector copies by ddPCR revealed that the average vector copy per cell in the dim populations ranged from 0.62–1 (Fig 5C). For all constructs, there was a higher average vector copy per cell in the bright population (Fig 5C), which was consistent with our earlier data showing a relationship between vector copy and MFI (Fig 4D). When we normalized average MFI for vector copy per cell in the bright and dim populations we found there were not consistent differences between constructs designed to reduce mispairing and the RH construct (Fig 5D). These indirect assessments of potential impact of mispairing on cell surface expression did not demonstrate its occurrence, nor did they exclude its possibility.

## Discussion

TCR based engineered cell therapy has shown its potential in cancer and antiviral settings, as underscored by the recent FDA approval of TECELRA (afamitresgne autoleucel), a treatment for advanced MAGE-A4+ synovial sarcoma in adults with particular HLA alleles. TCR based cell therapy success is predicated in part on sufficient cell surface expression of the introduced TCR. Transferring a therapeutic TCR of the same species may result in mispairing with the endogenous TCR, potentially reducing surface expression of the TCR of interest. To circumvent this phenomenon, a variety of approaches have been tested to promote preferential pairing of engineered TCRαβ combinations [62]. These approaches must balance the goal of increasing the amount of introduced TCR complexes expressed on the cell surface while reducing possible host immune responses to foreign TCR sequences. Additionally, modifications to the constant region alone are preferred, as they can be universally applied to TCRs of varying specificities.

One of the early strategies to improve therapeutic TCR surface expression was to use murine constant regions in human TCRs, known as “murinization”. This resulted in a higher surface expression and functional avidity of TCRs but came at the risk of causing an immunogenic response due to the foreign mouse segments in the TCR protein [34,63]. In an attempt to find a “Goldilocks” solution, Sommermeyer and Uckert identified a minimal set of amino acids within the α and β murine TCR constant chains that stabilized and enhanced murine TCR expression [45]. When the human TCR constant regions were mutated to express this minimal set of amino acids, the resulting “minimal murine (mm)” TCR was expressed at higher levels on primary human T cells and had enhanced TCR functionality compared to the wildtype human TCR. While there was clear improvement over wild type human TCR, these minimal changes did not enhance surface expression to the level of fully murinized constant region TCRs.

Given the complexity of cellular immunotherapy approaches, robust preclinical models are valuable tools to optimize engineering designs and methods and evaluate the potential of off-target tissue injury [64]. Nonhuman primates are an attractive candidate for such models as their physiology, genetics, and immune cell populations are highly similar to those of humans. Rhesus macaques are one of the most common nonhuman primate species used in biomedical research and have proven to be an indispensable model for the field of immunology, as exemplified by infection with SIV as a model for HIV and AIDS.

Beyond their suitability for studying a variety of infectious diseases, nonhuman primates are also susceptible to virus-induced cancers. Rhesus rhadinovirus (RRV) is related to Kaposi’s sarcoma-associated herpesvirus (HHV8) [65] and is associated with retroperitoneal fibromatosis that resembles Kaposi’s sarcoma in SIV-infected rhesus macaques [66]. Rhesus lymphocryptovirus (rhLCV) is highly homologous to human Epstein-Barr virus (EBV) and can cause B-cell lymphomas and hairy leukoplakia in SIV-infected macaques [67]. An oncogenic rhesus specific papillomavirus (RhPV-1) has been identified and shown to be sexually transmissible with oncogenic potential similar to high risk HPVs in humans [68].

Incidence and prevlance rates of cancer in rhesus macaques are difficult to accurately ascertain as many animals are euthanized for study endpoints prior to geriatric age when cancer is more likely to arise. However there is a significant increase in cancer incidence in animals older than 20 years, with neoplasia involvement in more than half of all documented deaths in rhesus macaques older than 26 years [69]. In addition to virus associated cancers, recent studies have shown increased rates of colorectal cancer in related rhesus macaques [70,71], similar to the autosomal dominant inheritance of human hereditary non-polyposis colorectal cancer [72,73]. Current efforts are underway to link phenotypes in large pedigreed rhesus macaque colonies to full length DNA sequences to detect naturally occurring genetic variations that may be inherited risk factors for cancer, such as BRCA1/2 mutations in humans [74].

Rhesus macaques used in infection models or those developing neoplasias provide an opportunity to study cellular immunotherapies in a treatment naïve and controlled setting. Use of these models for preclinical testing circumvents the need to recruit patients with late-stage disease progression who have typically undergone multiple treatment regimens and/or have treatment resistant disease, which may not accurately reflect outcomes of untreated patients who undergo cellular immunotherapy as a first line therapy. Given the potential power of these animal models for cellular immunotherapy, we wished to investigate methods to improve TCR cell surface expression due to its key role in cellular immunotherapy success. We extended previous work modifiying the TCR constant regions to a rhesus macaque system, by comparing a human codon optimized rhesus macaque SIV specific TCR (CM9 RH) with four alternative TCR constructs encompassing various approaches to enhance TCR expression (Fig 1). Our CM9 RH CYS TCR construct retains rhesus macaque α and β constant chains but creates a disulfide bond between the two chains by introducing cysteines at TRAC aa 48 (T) and TRBC aa 57 (S). The CM9 RMM (rhesus minimal murine) TCR contains rhesus macaque α and β constant regions with a minimal set of changes (TRAC aa 89–92 (T-E-S-V to S-D-V-P) and TRBC aa E17K, I21A, F132I, E135A, and Q138H) based on the findings of Sommermeyer and Uckert [45]. The CM9 MC TCR is a murinized rhesus TCR in which the TRAC and TRBC sequences are entirely murine with a disulfide bridge addition. Finally, we created the CM9 MMR (mouse minimal rhesus) TCR using the murine TRAC and TRBC sequences with TRBC aa93-112 replaced with the corresponding rhesus sequence to avoid predicted immunogenicity to this site [43].

We found that all four novel TCR constructs were expressed at the cell surface and that triggering of the transduced TCRs with MHC-presented cognate peptide induced specific responses in an intracellular cytokine staining flow cytometric assay. When rhesus primary macaque cells were transduced with equivalent amounts of γ-retroviral vector containing each of the five TCR constructs we observed a linear correlation between the percentage of cells expressing the CM9 TCR and the average number of cell associated vector copies. Similarly, there was a linear correlation between the MFI of CM9 tetramer staining and the average number of cell associated vector copies. To indirectly evaluate whether mispairing was reduced with alternative TCR constant regions we sorted transduced cells into negative, dim, and bright populations based on CM9 tetramer MFI and quantified cell associated vector copies for each population. We hypothesized that CM9 RH TCR transduced cells would be overrepresented in the dim MFI population relative to the other constructs, due to mispairing resulting in fewer correctly paired transduced CM9 TCRs being expressed at the cell surface. Our results were consistent with this theory, with CM9 RH TCR transduced cells having a greater proportion of cells falling within the dim gate compared to the other four constructs. The fully murinized construct had the lowest proportion of cells in the dim gate. We then quantified the average cell associated vector in the dim and bright populations to determine if more transduced TCR copies were required in RH TCR transduced cells in the bright population to overcome mispairing. While RH TCR transduced “bright” cells did have a higher average vector copy per cell compared to “dim” cells, this was true of all constructs, which was consistent with our data showing a relationship between vector copy and MFI. We did not observe a clear difference between average MFI per vector copy number for cells expressing constructs predicted to have greater or lesser impact of potential mispairing on cell surface expression of the transduced TCR, but our analysis may have lacked the resolution to demonstrate such a relationship if present.

Our data showed little difference between the RH and RH CYS TCR cell surface expression, suggesting that the addition of a disulfide bond between the two TCR constant chains did not significantly improve stability and expression. These results are consistent with other studies showing that mispairing is not eliminated with the disulfide bridge formation [37]. The fully murinized (MC) TCR consistently had the highest transduction efficiency and surface expression, as measured by CM9 tetramer staining, relative to the other TCR constructs tested. This is consistent with results from similar experiments with murinized human TCRs that showed enhanced expression on human primary cells [34,63]. Although inclusion of murine constant regions imparts clear benefits in terms of expression, it comes with the greatest risk of immunogenicity. The rhesus minimal murine (RMM) and murine minimal rhesus (MMR) are composed of chimeric constant regions that we hypothesized would retain the enhanced TCR expression of the MC construct, while reducing the potential for an immune response to a foreign protein. We found that both TCR constructs were capable of being expressed at the cell surface and retained functionality, as measured by cytokine responses to antigenic stimulation. Both constructs exhibited a similar level of cell surface expression, which was greater than the RH TCR, although not as high as the MC TCR. Given these promising results, further testing of the RMM and MMR TCR constructs to determine their feasibility for use in rhesus macaque models, including their potential for eliciting host immune responses, is of interest.

A limitation of this study is that we did not directly measure mispairing between the transduced and endogenous TCR α and β chains. Assays to directly measure mispairing are available and involve differentially tagging the N termini of the α and β chains. Each chain can be delivered individually, and the amount of the tag measured at the cell surface by flow cytometry is indicative of mispairing as the only mechanism for the chain to reach the surface is by mispairing with the complementary endogenous chain [45,75]. Alternatively, both tagged chains can be transduced into cells, labeled with fluorophore conjugated antibodies, and a measurement of FRET efficiency can be used to compare the likelihood of transduced constructs to preferentially pair [35]. While these approaches to directly measure TCR α and β chain mispairing are of interest, they were beyond the scope of the current study.

A caveat to this work is that our quantification of cell associated vector copies is averaged across total cell number, rather than on a per cell basis. This means that in a bulk population the average cell associated vector copy number will be underreported due to the presence of untransduced cells in the population. We addressed this limitation in part by quantifying the average cell associated vector copy number in TCR positive sorted populations as opposed to the alternative approach of single cell sorting TCR positive cells and expanding the cell clones for ddPCR quantification of cell associated vector copies.

The four alternative TCR constant region designs we have described are not the only strategies for improving cell surface expression. Two other approaches of note pursued by Bethune et. al [75] using a melanoma specific human TCR involve swapping constant domains between the α and β chains in the transduced TCR construct (domain swapped, “ds”) or replacing the αβ TCR constant domains with the corresponding γδ TCR domains. Both strategies reduced mispairing with endogenous TCRs while retaining antigenic specificity and functionality and would be interesting to pursue in a rhesus macaque model.

Beyond modifications to the TCR itself, engineering methods can reduce the potential of mispairing and improve cell surface expression of the desired TCR. In recent years, CRISPR/Cas9 technology has been applied to the cellular immunotherapy field, not only to regulate T cell differentiation and activation states, but also to knock out the enodogenous TCR and replace it with a TCR or CAR of interest [76–79]. This approach offers additional advantages as the TCR of interest is inserted at a specific site, rather than integrated semi-randomly, as is the case in lenti- or retroviral transduction, and it is expressed under the natural TCR promoter enabling physiological transgene regulation. The feasibility of this non-viral method of TCR engineering has been validated in two recent clinical trials [80,81].

In conclusion, we have identified multiple modifications that can be made to rhesus macaque TCR constant regions to improve cell surface expression of correctly paired TCR.

These results should inform the design of TCRs selected for use in rhesus macaque models of TCR based cellular immunotherapy to optimize high cell surface expression and functionality of the transferred TCR while reducing the likelihood of an immunogenic response that could cause the clearance of therapeutic cells.
